# Laboratory Exposure to Influenza A H2N2, Germany, 2004–2005

**DOI:** 10.3201/eid1212.060664

**Published:** 2006-12

**Authors:** Annette Schrauder, Brunhilde Schweiger, Udo Buchholz, Walter Haas, Daniel Sagebiel, Adrienne Guignard, Wiebke Hellenbrand

**Affiliations:** * Robert Koch Institute, Berlin, Germany

**Keywords:** Influenza A H2N2, laboratory, infection, quality assurance sample, influenza, case-control study, letter

To the Editor: From November 2004 to February 2005, a company contracted by the College of American Pathologists (CAP) sent influenza quality assurance samples containing live influenza A H2N2 viruses (A/Japan/305/57) to 3,748 international laboratories (*1**,**2*). Of these, 3,686 (98%) were located in Canada or the United States. In Germany, 6 laboratories received at least 1 set of 3 samples, 2 for virus antigen detection and 1 for virus culture; all contained live virus and were formatted identically. No information on infectivity or virulence of the samples was available. Because of the absence of human-to-human influenza A H2N2 virus transmission since 1968, this situation provided the rare opportunity to investigate whether infections with this strain had occurred in any of the laboratories.

We used a standardized questionnaire to obtain from the laboratories information on when the CAP samples had been received, which of the 3 quality assurance specimens they contained, and how many employees had been involved in their handling. A second questionnaire was distributed to personnel in microbiology and virology departments. This elicited information regarding routine laboratory activities, contact with CAP samples, tasks performed in conjunction with handling of the samples, and any influenzalike symptoms (sudden onset of fever, cough, headache, and muscle pain) within the respective time frame. Persons who had worked in a receiving laboratory from September 1, 2004, to April 15, 2005, and had performed routine procedures in virology (defined as transport of samples, preparation of samples for diagnostic testing, antigen testing, nucleic acid amplification testing, and virus isolation) were eligible for the study. From May 4 to May 19, 2005, we visited the laboratories to interview supervisory personnel regarding routine work-up of samples and to collect blood from study participants for serologic testing.

The national reference laboratory for influenza at Robert Koch Institute performed serologic testing for antibodies against A/Singapore/1/57(H2N2) virus by hemagglutination inhibition. We compared antibody titers of laboratory workers who worked with a CAP sample with those who did not. However, this comparison might not show a difference if (silent) virus transmission had occurred among laboratory staff. To exclude this possibility, we also compared titers of workers born before 1969 with those in a group of volunteers from Robert Koch Institute also born before 1969. Titers <10 were assigned a value of 1.

Of 47 laboratory workers, 18 either declined to participate or were excluded because they did not perform any of the defined routine procedures during the defined period. Thus, 29 (62%) workers were included in the study, of whom 14 (48%) reported having worked with CAP samples. Of these 14 workers, 12 (2 exclusively) transported samples and 11 (2 exclusively) prepared the samples; 9 (1 exclusively) performed antigen testing, 2 (0) performed nucleic acid amplification tests, and 4 (0) performed virus isolation. Fourteen workers (48%) reported no contact with the samples, and 1 (3%) was unsure. None of the 29 participants reported any event that could have led to release of infectious material during the respective time frame, such as broken test tubes or dropped culture plates. Participating laboratories reported that all procedures were performed under appropriate hygienic and safety precautions. No person had >3 symptoms typical for influenzalike illness in the 4 days after having worked with a CAP sample.

Specific influenza A H2N2 antibody titers were determined in 25 study participants. None had a titer >80, two (8%) had a titer of 80, three (12%) had titers of 40, two (8%) had titers of 20, and the remaining 18 (72%) had titers <10. Three (21%) of 14 workers exposed to CAP samples and 4 (40%) of 10 who denied exposure had titers >20. All 7 were born before 1969. The geometric mean of titers of all participating workers born before 1969 did not differ significantly from that of the Robert Koch Institute employees (p = 0.28; Figure).

**Figure:**
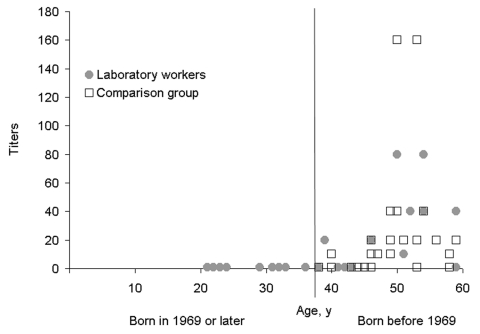
Titers of antibodies to influenza A H2N2 virus in laboratory personnel (n = 25; 13 born before 1969) and a comparison group born before 1969 (n = 32). The age listed is that in 2005. Titers <10 were assigned a value of 1.

In summary, no evidence was found for laboratory infections with the influenza A H2N2 virus. The risk for laboratory-acquired influenza infections is unknown. Severe acute respiratory syndrome coronavirus and Mycobacterium tuberculosis are infectious agents whose transmission characteristics are similar to those of influenza. For severe acute respiratory syndrome coronavirus, laboratory-acquired infections are well documented (*3**,**4*). For M. tuberculosis, there are strong indications that they occur (*5**–**7*). From a public health perspective and in view of the current importance given to influenza and a possible pandemic, accurate characterization of the risk for influenza infections in laboratory settings is needed. The small number of persons included in this study limits the conclusions that can be drawn. Potentially exposed workers were presumably tested in other laboratories involved, but we are not aware of any publications to this end. The lack of evidence for laboratory-acquired infection with A H2N2 in our study suggests that the risk was low under controlled laboratory conditions. However, only a large-scale serologic study (which might still feasibly be undertaken) could further substantiate this finding.
